# Deep Learning Diagnostics of Gray Leaf Spot in Maize under Mixed Disease Field Conditions

**DOI:** 10.3390/plants11151942

**Published:** 2022-07-26

**Authors:** Hamish A. Craze, Nelishia Pillay, Fourie Joubert, Dave K. Berger

**Affiliations:** 1 Centre for Bioinformatics and Computational Biology, Department of Biochemistry, Genetics and Microbiology, Forestry and Agricultural Biotechnology Institute (FABI), University of Pretoria, Pretoria 0028, South Africa; u15030335@tuks.co.za (H.A.C.); fourie.joubert@up.ac.za (F.J.); 2 Department of Computer Science, University of Pretoria, Pretoria 0028, South Africa; npillay@cs.up.ac.za; 3 Department of Plant and Soil Sciences, Forestry and Agricultural Biotechnology Institute (FABI), University of Pretoria, Pretoria 0028, South Africa

**Keywords:** deep learning, plant pathology, maize, gray leaf spot, *Cercospora*, field conditions, crop disease

## Abstract

Maize yields worldwide are limited by foliar diseases that could be fungal, oomycete, bacterial, or viral in origin. Correct disease identification is critical for farmers to apply the correct control measures, such as fungicide sprays. Deep learning has the potential for automated disease classification from images of leaf symptoms. We aimed to develop a classifier to identify gray leaf spot (GLS) disease of maize in field images where mixed diseases were present (18,656 images after augmentation). In this study, we compare deep learning models trained on mixed disease field images with and without background subtraction. Performance was compared with models trained on PlantVillage images with single diseases and uniform backgrounds. First, we developed a modified VGG16 network referred to as “GLS_net” to perform binary classification of GLS, which achieved a 73.4% accuracy. Second, we used MaskRCNN to dynamically segment leaves from backgrounds in combination with GLS_net to identify GLS, resulting in a 72.6% accuracy. Models trained on PlantVillage images were 94.1% accurate at GLS classification with the PlantVillage testing set but performed poorly with the field image dataset (55.1% accuracy). In contrast, the GLS_net model was 78% accurate on the PlantVillage testing set. We conclude that deep learning models trained with realistic mixed disease field data obtain superior degrees of generalizability and external validity when compared to models trained using idealized datasets.

## 1. Introduction

Crop diseases pose a serious threat to global food security [1]. Disease identification methods that function well outside of the lab are needed to correctly identify diseases and prevent instances of incorrect chemical control [1]. Crop disease monitoring by image analysis using a hand-held device such as a mobile phone is a goal of precision agriculture [2]. Such a tool could be provided free to resource-limited smallholder farmers [3]. It could also aid in high throughput phenotyping for rapid breeding of resistant crop varieties [4].

Gray leaf spot (GLS) is caused by the foliar fungal pathogens *Cercospora zeina* or *Cercospora zeae-maydis* that can be responsible for significant yield losses [2]. It presents as small, rectangular, matchstick-like lesions that expand parallel to the leaf vein and rarely, if ever, cross it [3]. These lesions start off as small yellowish discolorations on the leaf surface and gradually shift to a grayish-brown hue as the disease progresses.

Deep Learning (DL) is a technology that began gaining popularity in the late 1990s [4,5,6], which enables the identification of features inside dynamic environments. The applications of DL are wide and varied. They have seen use in audio denoising [7], land classification from satellite images [8], self-driving cars [9], drone detection [10], and more. Over the past decade, DL has also been used for plant stress phenotyping, primarily using image data [11,12].

Most attempts to use DL to identify plant disease (Table 1) do so using datasets generated under highly controlled lab conditions such as PlantVillage [13]. These datasets typically lack the complications endemic to the field and omit confounding features such as insect damage, multiple diseases per leaf, coalescing lesions of the same or different diseases, varied backgrounds, heterogenous lighting conditions, foreign objects in a frame such as hands and feet, and so on. When models trained on these controlled datasets are asked to perform outside of ideal conditions, they tend to perform poorly [13,14].

There are a few papers that investigate DL and related approaches for disease identification in maize leaves [15,16,17,18,19,20]. However, most of these papers make use of PlantVillage images, apart from a series of studies on northern corn leaf blight (NCLB) detection in maize field trial images where only this disease was prevalent due to artificial inoculation [16,20,21].

**Figure 1:**
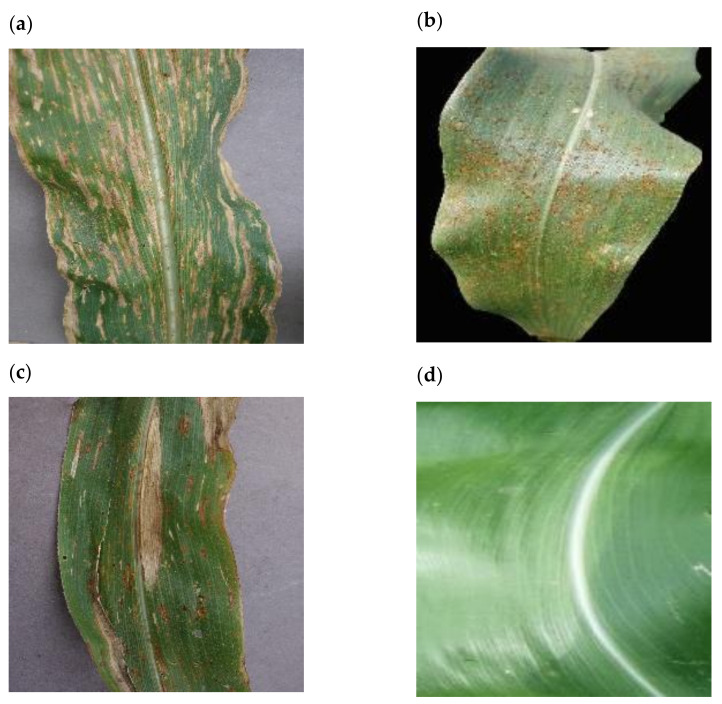
Images of maize leaves obtained from PlantVillage. (**a**) Image labelled as GLS positive. (**b**) Image labelled as CR positive, note the presence of Phaeosphaeria Leaf Spot (PLS). (**c**) Image labelled as NCLB positive, note the presence of CR. (**d**) Image labelled as ‘Healthy’.

The PlantVillage dataset [13] also contains several confounding features that impact the generalizability of models trained using this data. Namely, both GLS and NCLB had grey backgrounds (Figure 1a,c), all images with Common Rust (CR) had a black background (Figure 1b), and all healthy images had no background (Figure 1d). In effect, this means that models trained using these images could make predictions based on the presence or absence of background pixels alone.

No approach could be found that accounts for the range of complexity in crop fields. There are three reasons thought to contribute to this: (i) the difficulty of generating a dataset of sufficient size and complexity; (ii) the need for plant pathology experts to label sufficient numbers of images for training the DL models; and (iii) datasets that produce high accuracies are more likely to be popularized than those that produce less useful results.

There is limited use in digital plant pathology of validation algorithms to confirm that DL models do indeed detect disease symptoms in images, for example, tools such as Grad-CAM [22] and Grad-CAM++ [23]. These algorithms produce heatmaps that overlay images that correlate with regions associated with class activation by a DL model. The heatmaps must be manually inspected and thus this approach is impractical at scale. However, they are vital in aiding explainability.

Image backgrounds are known to impact model performance [24]. Some researchers may opt to remove the background from their images via segmentation tools such as GrabCut [25,26]. These interventions require human input which, as with Grad-CAM, limits their applications at scale. Some practitioners have used DL models in a pre-processing step to remove background from images, using tools such as MaskRCNN [27,28,29].

The main shortcomings we identified in the field of artificial intelligence-based crop disease identification were the lack of research that makes use of in-field data in realistic conditions and a lack of methodologies that address the poor generalization exhibited by models trained on lab-based image datasets. There is limited research that accounts for multiple disease symptoms on one leaf. There are few papers that make use of explainability tools that report which pixels of the image were detected as a positive identification by the DL model [38]. We aimed to address this by demonstrating how models perform under realistic (i.e., uncontrolled) conditions versus idealized conditions. In addition to this, we also propose a method for segmentation using a MaskRCNN network and investigate the effect of background removal on model performance.

To our knowledge, this paper is the first contribution to the DL-driven identification of GLS in maize with mixed diseases under field conditions. In this work, it is shown that DL is capable of scaling outside of lab conditions, provided that sufficient data can be made available.

## 2. Materials and Methods

### 2.1. Datasets

Two data sources were used. The first was developed for this study and contained images of maize leaves with and without disease symptoms, obtained in field conditions (IF). The second was the subset of maize leaf images from PlantVillage [13]. The IF dataset was in turn used to generate two additional datasets, the IFL and the IFNB. Each dataset is described below.

#### 2.1.1. IF Dataset

The “In-field” dataset (IF) contains a total of 2332 maize leaf images. Images were obtained from a variety of maize field locations where foliar diseases are prevalent in Mpumalanga, KwaZulu-Natal, and Eastern Cape Provinces, South Africa (Berger et al., 2020). Devices ranging from smartphones to a Nikon D90 SLR camera (Tokyo, Japan)were used to capture the images. All images were resized to 224 by 224 pixels across the dataset to reduce training times and ensure homogeneity across all images. The IF dataset is available on Kaggle [39].

**Figure 2:**
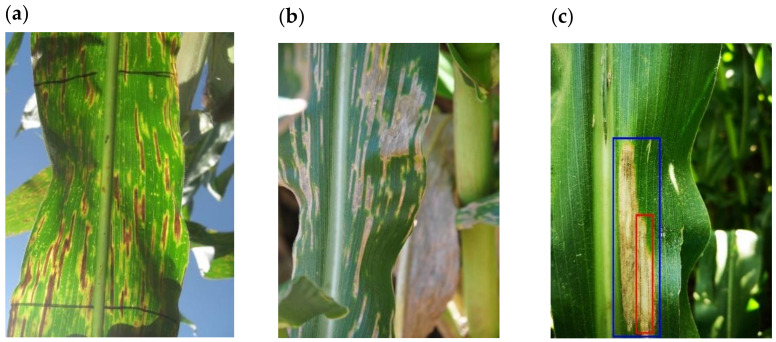
Example images of GLS symptoms on maize leaves in the In-field (IF) dataset. (**a**) Example of GLS lesions as visualized from under the leaf. (**b**) Example of GLS coalescing into larger, differently shaped lesions. (**c**) Example of a GLS lesion (red) occurring inside an NCLB lesion (blue).

Each image was manually inspected and labelled for the presence of maize foliar diseases by plant pathologists familiar with the disease symptoms. The identities of the disease-causing fungi were confirmed for some samples by microscopy and molecular methods, namely GLS caused by *C. zeina* (Nsibo et al., 2019) (Figure 2), NCLB caused by *Exserohilum turcicum* (Berger et al., 2020), and CR caused by *Puccinia sorghi* (Berger et al., 2020). Labelled images could contain one or more disease classes simultaneously. During image capture, it was commonly noted that multiple diseases could co-occur and even coalesce into unique and novel formations. For example, a GLS lesion was observed in one case inside a larger NCLB lesion (Figure 2c).

Table 2 gives a breakdown of the labelled dataset. Table 3 contains a breakdown of the extent of disease co-occurrence within the dataset.

During training, the IF dataset was artificially augmented through mirroring and rotation at 90 degrees, which increased the dataset 8-fold to a total of 18,656 images.

#### 2.1.2. IFL Dataset

**Figure 3:**
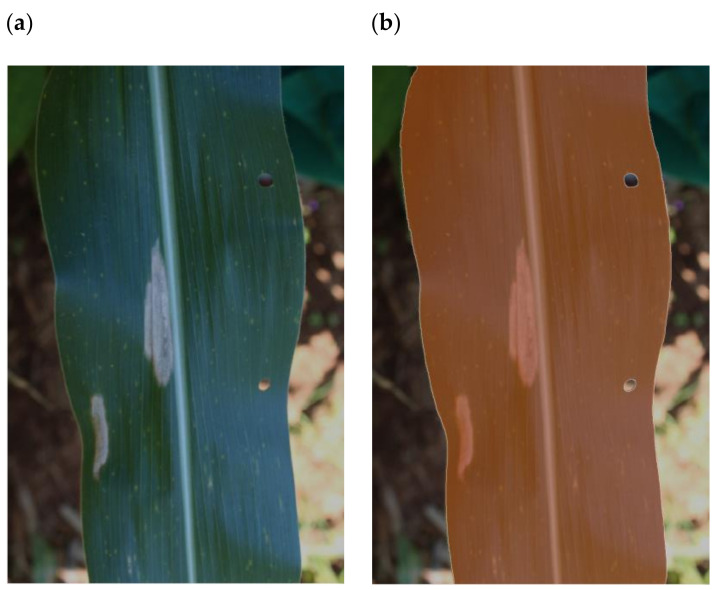
Example of image segmentation to define the leaf area for the “In-field_leaf” (IFL) dataset. (**a**) Original leaf image from the In-field (IF) dataset. (**b**) Leaf area from image (**a**) highlighted manually and shown by brown overlay using the tool available at https://segments.ai (accessed on 21 July 2022).

The “In-field_leaf” dataset (IFL) is a subset of the 2332 images in the IF dataset. It contains 615 randomly selected images. These images were manually segmented using the online tool segments.ai [40], in a manner shown in Figure 3. Only the single most prominently displayed leaf was selected by the annotators. These images were later used to train a leafRCNN network (described in Section 2.2.1).

#### 2.1.3. IFNB Dataset

**Figure 4:**
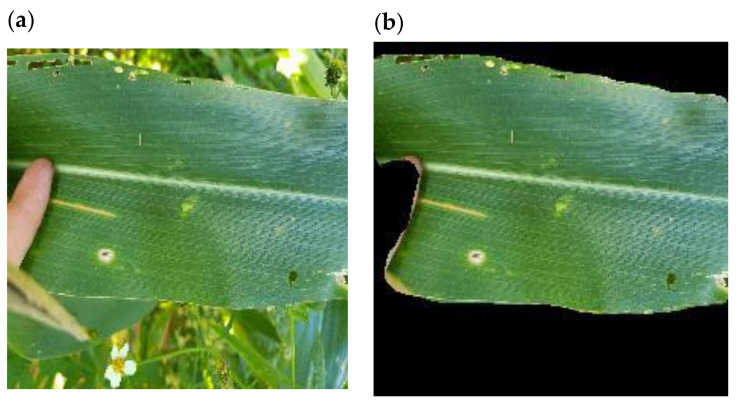
(**a**) Image of a maize leaf and (**b**) the same leaf after leafRCNN leaf area prediction and background removal.

The “In field_noBackground” dataset (IFNB) is a one-to-one variant of the IF dataset (including augmentation) where the leaves have been extracted from their backgrounds (Figure 4). Leaf masking was performed by a custom MaskRCNN network referred to here as leafRCNN. leafRCNN was used to identify the area of the maize leaf. The resulting mask was then used to “extract” the leaf from the background by setting all non-leaf pixels to 0.

#### 2.1.4. PlantVillage Dataset

PlantVillage (PV) is a large publicly available dataset consisting of some 54,303 images across 38 class labels [13,41]. Of that, 3852 are maize images. This is further split between Gray Leaf Spot (GLS), Northern Corn Leaf Blight (NCLB), Common Rust (CR), and Healthy classes (Table 4). PLS and SR diseases are not labelled in the PV dataset.

PlantVillage uses a crop-class pairing strategy to label its images. A single image will belong to a single crop and will correspond to a single disease class. Images that present with multiple diseases will only receive a single disease label. In cases where there are simultaneous disease classes on a single leaf, we were not able to determine how the main disease class was selected. This means that there are images within the PV maize subset that possess a combination of GLS, NCLB, and CR but are labelled with only one disease.

### 2.2. Model and Training

All models were created using *PyTorch* and trained on an Nvidia V100 (Santa Clara, CA, USA) with 16GB of RAM at the CSIR Centre for High Performance Computing (CHPC) in Cape Town, South Africa. Models were trained using a regime which consisted of five separate runs, each of which contained 10 epochs. Weights were initialized using pretrained values from ImageNet. Models were reinitialized at the start of each run. A training/testing split of 75%/25% was used for all datasets. At the end of each epoch, a model’s performance on its respective testing set was assessed through loss metrics. This was then used to select the best model across all runs and epochs. A breakdown and description of all the models used are detailed below.

#### 2.2.1. leafRCNN

leafRCNN was developed as a MaskRCNN [29] network pretrained on ImageNet [42]. The MaskRCNN was adapted from Pytorch’s native implementation, and a custom classification layer was added. It was trained using the IFL dataset and no data augmentation was applied. leafRCNN produces a mask that corresponds to leaf area. This mask was then used in combination with python packages *NumPy* and *pillow* to set non leaf pixels to 0, thus “segmenting” the image. MaskRCNN uses several loss metrics, these include loss_classifier, loss_box_reg, loss_mask, and loss_objectness [29]. These loss values were summed and averaged, and the resultant loss was used during training and backpropagation. A batch size of two was used during training.

#### 2.2.2. GLS_net

GLS_net was developed as a modified VGG16 CNN pretrained on ImageNet. The network was implemented using PyTorch’s native implementation and the default classifier was switched in favour of a custom classifier intended for binary classification (GLS, notGLS). A learning rate of 0.0001 was selected and ADAM [43] was used as the optimizer (betas = (0.63,0.968), and eps = 1 × 10^−7^; these values were obtained from hyperparameter tuning, the details of which are not discussed here). The loss was calculated using Binary Cross Entropy for GLS_net and the models derived from it using different datasets. GLS_net was trained using the IF dataset using a batch size of 64. The performance metrics calculated were accuracy, precision, recall, and F1 score. After training, the best GLS_net model was selected based on the best (lowest) loss across all runs and epochs of the IF testing set. This model was then used to predict the unseen PV testing set. The model’s performance across the IF and PV testing sets was then compared.

#### 2.2.3. GLS_net_pv

This model was identical in architecture to GLS_net, with the exception that it was trained using the PV training set using a batch size of 64. After training, GLS_net_pv was then used to predict upon the IF testing set (without any training). The model’s performance across the PV and IF testing sets was then compared (accuracy, precision, recall, and F1 score).

#### 2.2.4. GLS_net_noBackground

This model was identical in architecture to GLS_net, with the exception that it was trained using the IFNB training set using a batch size of 64. After training, GLS_net_noBackground was asked to predict upon the testing set of the PV dataset. The model’s performance across the IFNB and PV testing sets was then compared (accuracy, precision, recall, and F1 score).

### 2.3. Visualization

Two explainability tools, described below, were used. These tools produce heatmaps that correspond to areas associated with high class activations by the CNN model being tested. Using the IF dataset and a trained GLS_net, both tools were used to provide an intuitive glimpse into the difference in performance observed between GLS_net and GLS_net_noBackground.

#### 2.3.1. Grad-CAM

Grad-CAM is a tool used for “visual explanations” of CNN networks [22]. It produces heatmaps that when overlayed atop the original image, will correspond to areas of the image that were significant in the prediction of the output class. A paper titled “Sanity Checks for Saliency Maps” reports on the investigation of a number of visualization tools including Grad-CAM [44]. Grad-CAM was one of the few visualization tools investigated that passed the authors’ “sanity checks”.

#### 2.3.2. Grad-CAM++

Grad-CAM++ is a variant of Grad-CAM [23]. According to the paper, Grad-CAM may produce erroneous or poorly interpretable heatmaps when more than a single object is present in an image. Grad-CAM++ claims to improve upon this weakness and thus was selected as an additional tool for visualisation due to the multiple disease lesions within many of the images in the dataset.

## 3. Results

### 3.1. Identification of GLS Disease in Mixed Disease Images (“GLS_net” CNN)

A deep learning CNN named GLS_net was developed using the In-field (IF) dataset of 2332 images (augmented to 18,656 images), where 46% of the images had symptoms of GLS disease. Training was conducted on 75% of the images from the IF dataset (13,992). After training, the best model was selected based on it having the best (lowest) loss when applied to the testing set (the remaining 4664 images of the IF dataset). This “best” model achieved a 75.3% accuracy on the IF training set. Best loss values for each model of GLS_net applied to the testing set were compared to determine if there were any outliers. GLS_net achieved an average best loss for the testing set of 1.01 and a standard deviation of 0.0061 across all runs (Figure S1). This indicated that the models were tightly clustered across each run with no outliers. The version of GLS_net with the best loss was selected for subsequent assessment of the testing sets.

Table 5 and Table 6 show a breakdown of the performance achieved by GLS_net upon the IF and PV testing sets. GLS_net performed well in identifying GLS disease in the mixed disease (IF) testing dataset (4664 images) with an accuracy of 73.4% (Table 5). Accuracy was calculated as the number of images correctly identified as containing GLS or not, divided by the total images tested (Table 5). Furthermore, although GLS_net was not trained using images from PV, it yielded an accuracy of 78.6% (Table 6), supporting its ability to identify the characteristic symptoms of GLS. In Table 5 (IF testing set), in 86% of cases of GLS_net predicting GLS, this reflected the ground truth (precision). However, GLS_net only finds 50% of all cases of GLS in the IF testing set (recall rate). This indicates that while GLS_net is not a sensitive model, it is a highly specific one.

GLS_net finds a higher proportion of GLS images in the PV testing set (65.7% recall rate, Table 5) than in the IF testing set, which indicates that the presentations of GLS within the PV testing set are more homogenous than those of the IF testing set. Precision by GLS_net in identifying GLS was much lower for the PV testing set (37.5%). This may be explained by two factors: (i) the PV dataset was not labelled with the idea of disease co-occurrence in mind, and (ii) PV contains mislabelled images (Section 2.1.4).

To estimate the extent of mislabelling, 100 random images were selected from the CR and NCLB subsets (2177 images) of the PV dataset. Manual inspection revealed 44 of the 100 images to also contain GLS symptoms, indicating an estimated false negative rate in the PV dataset of 44% (95% confidence interval [34%, 54%]). Therefore, single disease labelling and mislabelling may have impacted the precision seen in Table 6.

### 3.2. Identification of GLS Disease in Mixed Disease Images Using Model Trained on PlantVillage Images (“GLS_net_pv” CNN)

A deep learning CNN named GLS_net_pv was developed using the PV dataset of 3852 images, where 13% of the images were labelled as GLS disease. GLS_net_pv achieved an average best loss for the testing set of 1.01 with a standard deviation of 0.0061. The version of GLS_net_pv with the best loss (0.248) was selected for subsequent assessment (Figure S2).

Table 7 and Table 8 show a breakdown of the performance achieved by GLS_net_pv upon the PV and IF testing sets. Overall, performance was dissimilar between the two testing sets. GLS_net_pv achieved a 94.1% accuracy on the PV testing set, which is comparable with models trained with PV in previous studies (Table 1). However, when GLS_net_pv was asked to predict upon the less idealized IF dataset (Table 8), a drop-off in accuracy is seen (55.1%). This trend is consistent across all measure metrics.

Notably, recall of GLS_net_pv upon the IF testing set drops to 5.2% (Table 8). This indicates that models trained using PV are highly insensitive to GLS symptoms in mixed disease images that are often observed in the field. The IF testing set has a class balance of 46% GLS and 54% notGLS. GLS_net_pv achieves an accuracy of 55.1%, which is only marginally better than an accuracy achieved by a zero rule classifier (predicts based solely on class balance). The results indicated that models trained using PV failed to generalize outside of the PV dataset.

### 3.3. Development of a CNN to Extract the Leaf Area from an Image (leafRCNN)

We attempted to improve the accuracy of GLS disease identification in images by “removing” non-leaf background pixels. A subset of 615 images from the IF dataset was used to train a MaskRCNN model to identify the main leaf area in an image. The resultant model, named leafRCNN, was successful in identifying and localizing the main leaf body in an image (Table 9, Figure S3). Notably, leafRCNN was able to differentiate between leaves and obvious foreign objects such as hands and fingers (Figure 4b). leafRCNN was deemed adequate and was used to perform leaf and background segmentation across the remainder of the IF dataset (1717 images) to generate the IFNB dataset.

### 3.4. Identification of GLS Disease in Mixed Disease Images Using Model Trained on In Field Images without Background (“GLS_net_noBackground” CNN)

The deep learning CNN named GLS_net_noBackground was developed using the IFNB dataset of 18,656 images. After training models on the training set (75% of IFNB images), the models were compared using the testing set (25% of IFNB images). The best GLS_net_noBackground models achieved an average best loss of 1.0481 with a standard deviation of 0.0023 (Figure S4). This indicated that models were tightly clustered across each run. The best loss achieved was 1.0441. This version of GLS_net_noBackground was selected for subsequent assessment.

GLS_net_noBackground achieved an accuracy of 72.6% in identifying GLS disease in the testing set of images with the background removed (IFNB dataset) (Table 10). This was marginally worse than the 73.4% accuracy of GLS_net on the same set of images without background removal (IF dataset) (Table 5). The other performance metrics, such as precision and recall were also very similar between the two models (compare Table 10 with Table 5). Using a one-tailed t-test, it was found that the decrease in accuracy between GLS_net (M = 73.25, SD = 1.22) and GLS_net_noBackground (M = 72.27, SD = 0.16) was significant (t(8) = 1.8818, *p* = 0.048). These results indicate that training DL models with datasets where backgrounds have been removed do not significantly improve the identification of GLS disease.

GLS_net_noBackground was able to detect GLS disease in the PV dataset to a similar level of accuracy as GLS_net (76.1% and 78.6%, respectively), but also with low precision, likely due to the limitations of the PV dataset labelling as discussed in Section 3.1 (compare Table 11 with Table 6).

### 3.5. Visualization

**Figure 5:**
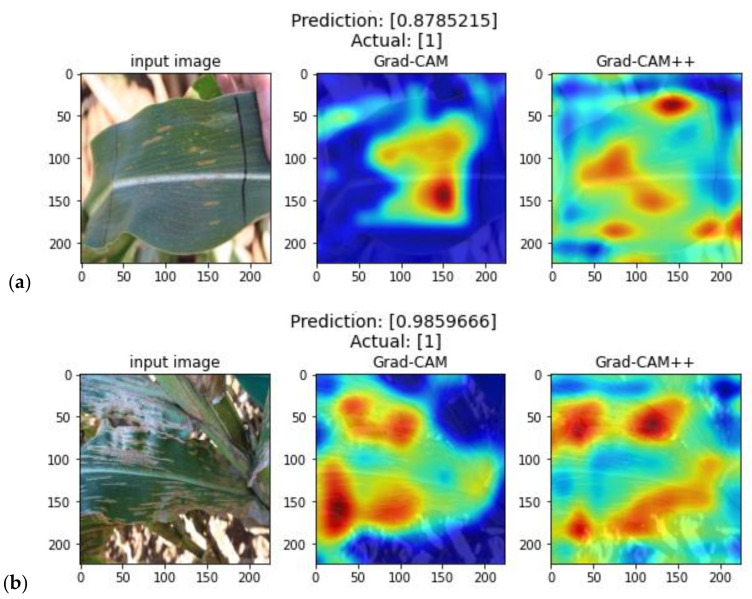

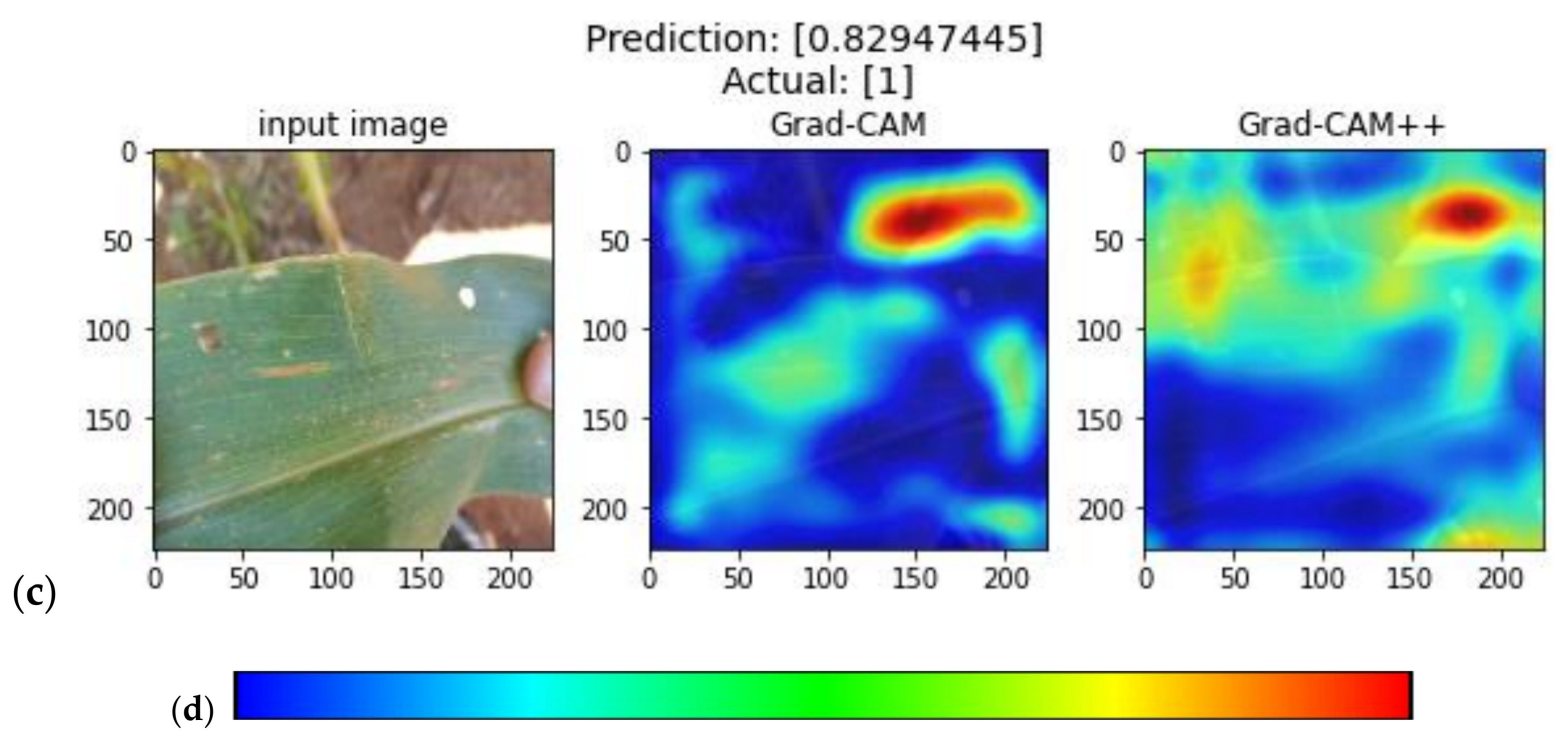
Heatmaps from Grad-CAM and Grad-CAM++ software, which are designed to illustrate image regions detected as GLS positive by a CNN, such as GLS_net, are shown here. Panels (**a**–**c**) show three GLS positive representative images from the IF dataset. Each panel shows (from left to right) the input image that was scored as GLS positive by GLS_net, the Grad-CAM heatmap, and the Grad-CAM++ heatmap, respectively. Panel (**d**) contains a colour scale to aid in interpretation, blue indicates no activation, while red indicates high levels of activation.

Figure 5 shows examples of heatmaps generated by Grad-CAM and Grad-CAM++ using the network activations of GLS_net on images from the IF dataset that contain GLS. Grad-CAM heatmaps in Figure 5a,b show activations around some of the GLS lesions. However, in Figure 5c the main activation corresponds to a bright region above the leaf edge, with a weak activation around the lesion on the leaf. Grad-CAM++ is purported to function better in scenarios where there are multiple instances of classes within a single image [23]. However, when applied to diseased maize leaf images, Grad-CAM++ heatmaps tended to activate in regions of high contrast on the images, such as the edges of leaves as can be seen in Figure 5c. These representative results indicate that the lack of good correlation between actual GLS lesions and heatmap activation may be due to problems with Grad-CAM in extracting the network activation data from these types of images.

## 4. Discussion

The main finding from this study was the development of a CNN named GLS_net, which could identify GLS disease symptoms on maize leaf disease images at an accuracy of 73.4%. Importantly, this accuracy was achieved from field images with symptoms of mixed diseases common in sub-Saharan Africa [45]. The main diseases in addition to GLS, which has thin matchstick-like lesions, were NCLB which has larger cigar-shaped lesions with pointed ends, CR which is characterized by reddish-brown pustules, and PLS with white spots [3,45,46]. The GLS_net CNN was developed using a relatively small dataset of 2332 images, but augmentation was used to increase the dataset 8-fold prior to training.

In this study, a second CNN named GLS_net_pv was trained using the PlantVillage maize disease dataset. This is a standardized dataset photographed in the lab against a homogenous background with single leaf images labelled as GLS, NCLB, CR, or no disease. GLS_net_pv achieved an accuracy of 94.1% on the PV testing set, which is similar to accuracies in the 90th percentile from previous deep learning models trained on the PV dataset [13,17,19,34,35]. However, GLS_net_pv performed poorly at identifying GLS in the field-derived mixed disease dataset with an accuracy of 55.1%, which illustrates the problem of applying a lab-image trained model to more complex field images. In contrast, the mixed-disease field image trained model GLS_net performed well in GLS identification in the PV dataset (78.6% accuracy) and the field disease dataset (73.4% accuracy). We conclude that (i) models can be trained using data obtained under realistic conditions and still provide reliable disease predictions; and (ii) these models are more robust and consistent across datasets. The accuracy of GLS_net is likely to increase as more images are added to the dataset. Hyper-parameter tuning is an additional approach that could be used to improve the GLS_net model [47].

Background removal has been considered a method to improve the performance of CNNs by removing confounding objects from images [27]. Field images of maize leaves with disease symptoms were thought to be good candidates for background removal since most images were made up of the main leaf in focus with different backgrounds. MaskRCNN has proven to be a useful tool for image segmentation [28,48]. In this study, it was adapted to produce a model called LeafRCNN which extracted maize leaves from their backgrounds. Importantly this was achieved with a relatively small subset of training images from the IF dataset, which were manually labelled using segments.ai [40] for ground-truthing. LeafRCNN was then used to automatically remove the background of the complete IF dataset. Alternative methods of background removal such as GrabCut are potentially more time-consuming since GrabCut requires more manual intervention to be performed effectively [49]. For this approach to work best, datasets should be comparatively homogenous, as is the case with the IF dataset where most images had a single leaf in focus.

Surprisingly, the removal of backgrounds from the maize mixed disease image set did not produce a CNN (GLS_net_noBackground) with better GLS identification than the CNN trained on the original images with backgrounds (GLS_net). GLS_net_noBackground had a 72.6% accuracy compared to GLS_net accuracy of 73.4% (Table 5 and Table 10). A possible reason may be that some networks employ contextual cues to perform classification. In this regard, Xiao et al. [24] noted that some models in their study were able to achieve “non-trivial accuracy by relying on the background alone”. However, background removal has proven useful in some cases in improving CNN performance [27,38]. Further research is required to determine why the removal of backgrounds around maize leaf disease field images did not result in significant improvements to the DL-based mixed disease identification.

In this study, versions of GradCAM [22,23] were employed to attempt to identify which regions of images were activated by the GLS_net CNN. It was found that the GradCAM heatmaps were activated in the correct areas of GLS lesions in some images, however, GradCAM++ did not perform well since it activated non-disease regions of high contrast on the images. GradCAM has been used previously to interrogate CNNs developed for plant disease images [38], however, performance was better for images where backgrounds had been removed. This indicates that further optimisation of validation tools is required to deal with complex subjects such as mixed disease images. Improved validation tools are required since it has been noted that implementing DL models in practise with a lack of explainability may hold ethical and legal implications [50].

Plant disease image datasets that have been used for training DL models for disease identification have to date been focused on single diseases on a single leaf, for example, PlantVillage (54,306 images for 14 plant species) [13] and the maize image database with NCLB images (18,222 images) [51]. Such public datasets are commendable and have been used by others for the development of single disease/single leaf DL models [15,20,30].

The goal of our study was to address the challenge of identifying GLS disease in field images where symptoms of more than one disease were present on one leaf, and thus we developed a custom dataset of 2332 images, which was increased to 18,656 by augmentation. We initially attempted to develop a GLS disease identification CNN (GLSnet_pv) using the PlantVillage dataset for training, however, the accuracy was not sufficient compared to the GLS_net trained on the more complex multi-disease dataset. This highlighted some of the limitations of lab image datasets such as PlantVillage. First, images are only labelled with a single disease, however, some leaves had additional disease symptoms (see Figure 1b,c for examples). Second, the maize no-disease images were zoomed in so that the leaf filled the image with no background, whereas most maize disease images showed leaf pieces with either a grey or black homogenous background. A CNN trained on this dataset to distinguish between maize disease and no-disease could achieve an inappropriately high level of accuracy based on the presence or absence of background pixels.

There is a need in the discipline of plant disease diagnosis to expand the current image datasets that are available for developing artificial intelligence solutions with deep learning. In this study, maize disease images were labelled for the presence or absence of different diseases by experienced field plant pathologists, a low throughput process. In addition, leaf areas were extracted using an online tool [40]. The bottleneck in generating useful datasets is labelling each image to indicate either (i) the presence/absence of a disease symptom; or (ii) segmenting each image to define the positions of disease symptoms. Segmentation is important for applications where disease quantification is required, such as in crop breeding for disease resistance [28,47]. Current image datasets have the limitation that they are static, and not updated. There is a need for a collaborative image database platform that is (i) open access, (ii) actively maintained and curated, and (iii) searchable.

## 5. Conclusions

This work addresses the challenge of automatically identifying a single maize leaf disease (gray leaf spot) in realistic field images where there is more than one disease type on a leaf image. Most previous attempts at applying artificial intelligence to plant disease identification were based on image datasets with a single disease per leaf, often with homogenous backgrounds (see Table 1). First, this work contributes a field-captured labelled dataset of 2332 maize leaf images with mixed disease symptoms [39]. Second, a deep learning (DL) model based on convolutional neural networks (GLS_net) trained on the field dataset was able to identify GLS disease at 73.4% in the field image testing set. This highlights the importance of training DL models with realistic field images, as it was a major improvement compared to the 55.1% accuracy of a DL model trained on the PlantVillage maize dataset (single disease, uniform background images). Third, pre-processing images by removing the background around the leaf (using a DL model leafRCNN) to produce a new training set did not improve the accuracy of GLS disease identification. Future improvements will include (i) a systematic approach to upscaling the number of mixed disease images in the training set based on the number of different disease classes; and (ii) an ensemble approach to identifying more than one disease in mixed disease images.

## Figures and Tables

**Table 1:** Deep learning applications for plant disease classification.

Plant Species	Disease	Dataset	Size	Architecture	Highest Accuracy	References
Apple	Black Rot	PlantVillage	2086	VGG-16, VGG-19, Inception-v3, ResNet50	90.4%	[30]
Maize	Northern Corn Leaf Blight	Manual *	1796	CNN	96.7%	[16]
Maize	Northern Corn Leaf Blight	Manual	3000	MaskRCNN	96% (AP) *	[20]
Maize	Common Rust	PlantVillage	1800	VGG-16	89%	[31]
Maize	Southern Leaf Blight, Brown Spot, Curvularia Leaf Spot, Rust, Dwarf Mosaic, Gray Leaf Spot, Round Spot, Northern Leaf Blight	PlantVillage and Various	500	GoogLeNet	98.8%	[15]
Maize	Common Rust, Gray Leaf Spot, Northern Corn Leaf Blight, Healthy	PlantVillage	3852	Modified LeNet	97.89%	[17]
Maize	Rust, Nothern Corn Leaf Blight, Healthy	Manual in Tandem with PlantVillage	4382	Custom DCNN	88.46%	[19]
Pear, cherry, peach, apple, grapevine	7 diseases (fungal, oomycete, bacterial, mites	Various	30,880	CaffeNet	96.3%	[32]
Potato	Potato Blight	PlantVillage	300	SVM	95%	[33]
Soybean	4 diseases (fungal, bacterial), 3 abiotic stresses	Manual, But Highly Controlled	6000	DCNN	94.13%	[34]
Tomato	One bacterial, two viruses, five fungal diseases, spider mites	PlantVillage	14,828	AlexNet, GoogLeNet and others	99.18%	[35]
Tomato	5 diseases (fungal, oomycete, bacterial), 2 insects, 2 abiotic factors	Manual	5000	Faster R-CNN, R-FCN, SSD	85.98%	[36]
Wheat	Powdery Mildew, Smut, Black Chaff, Stripe Rust, Leaf Blotch, Leaf Rust, Healthy Wheat	WDD2017	9230	VGG-FCN-VD16, VGG-FCN-S	95.12%	[37]
14 crops (dicots, trees monocots)	38 Diseases (fungal, oomycete, bacterial, viral)	PlantVillage	54,306	AlexNet, GoogLeNet	99.35%	[13]

* Manual = image dataset developed by authors. * AP = Average precision. MaskRCNN networks are not assessed using accuracy.

**Table 2:** Breakdown of disease classes found in the In-field dataset.

Disease	Total
Gray Leaf Spot (GLS)	1084
Northern Corn Leaf Blight (NCLB)	554
Phaeosphaeria Leaf Spot (PLS) *	493
Common Rust (CR)	300
Southern Rust (SR)	39
No Foliar Symptoms	285
Other	324
Unidentified	309
Total Images	2332
Total Disease observations	3388

* Also known as White Spot Disease.

**Table 3:** Extent of disease co-occurrence in the In-field (IF) dataset.

Number of Classes per Image	Total
1	1415
2	48
3	691
4	31
5	128
6	13
7	19
8	0
AVG number of classes per image	1.45
STD of number of classes per image	0.63

**Table 4:** Breakdown of classes found in the PV dataset.

Disease	Total
Gray Leaf Spot	513
Northern Corn Leaf Blight	1192
Common Rust	985
Healthy	1162
Total	3852

**Table 5:** Performance of GLS_net upon the IF testing set.

Name	Precision	Recall	F1-Score	Support
GLS	86.3	50.0	63.3	2136
notGLS	68.8	93.3	79.2	2528
Macro Avg	77.5	71.6	71.2	4664
Weighted Avg	76.8	73.4	71.9	4664
Accuracy	73.4			

Values are provided as percentages (%). Precision = TP/(TP + FP), Recall = TP/(TP + FN), Accuracy = (TP + TN)/(TP + TN + FP + FN). TP = true positive; FP = false positive; TN = true negative; FN = false negative. F1-Score = 2 × (Precision × Recall)/(Precision + Recall). Macro Avg: verage score of metric assuming equal weighting (cannot be calculated from this table, requires underlying data). Weighted Avg: Average weighted score of metric. Metrics are weighted according to class proportion (cannot be calculated from this table, requires underlying data). Support: The total number of images associated with the class.

**Table 6:** Performance of GLS_net upon the PV testing set.

Name	Precision	Recall	F1-Score	Support
GLS	37.5	65.7	47.7	143
notGLS	93.1	80.9	86.6	820
Macro Avg	65.3	73.3	67.1	963
Weighted Avg	84.9	78.6	80.8	963
Accuracy	78.6			

**Table 7:** Performance of GLS_net_pv upon the PV testing set.

Name	Precision	Recall	F1-Score	Support
GLS	89.8	67.8	77.3	143
notGLS	94.6	98.7	96.6	820
Macro Avg	92.2	83.2	86.9	963
Weighted Avg	93.9	94.1	93.7	963
Accuracy	94.1			

**Table 8:** Performance of GLS_net_pv upon the IF testing set.

Name	Precision	Recall	F1-Score	Support
GLS	61.2	5.2	9.7	2136
notGLS	54.8	97.2	70.1	2528
Macro Avg	58.0	51.2	39.9	4664
Weighted Avg	57.7	55.1	42.4	4664
Accuracy	55.1			

**Table 9:** leafRCNN performance upon the IFL testing set.

Metric	IoU Range	Score
Bbox Precision	0.50:0.95	99.0%
Bbox Recall	0.50:0.95	99.0%
Segm Precision	0.50:0.95	92.3%
Segm Recall	0.50:0.95	94.4%

Bbox = Bounding Box. MaskRCNN predicts bounding boxes where it believes instances to be contained within. These metrics track how well leafRCNN predicts bounding boxes that overlap with the ground truth (GT). Segm = Segmentation. MaskRCNN predicts masks that should overlay with GT labels. These metrics track how well these predicted masks overlap with GT. Precision = The average precision value obtained between multiple IoU values. Recall = The average recall value obtained between multiple IoU values. IoU = Intersection over Union. Measures the degree of overlap between two 2D objects. 0.50:0.95 indicates that the obtained Precision and Recall values were generated over a range of IoU values between 0.50 and 0.95 using a 0.05 step. Further metrics of leafRCNN performance are given in Tables S1 and S2.

**Table 10:** Performance of GLS_net_noBackground upon the IFNB testing set.

Name	Precision	Recall	F1-Score	Support
GLS	85.0	48.8	62.0	2136
notGLS	68.2	92.7	78.6	2528
Macro Avg	76.6	70.8	70.3	4664
Weighted Avg	75.9	72.6	71.0	4664
Accuracy	72.6			

**Table 11:** Performance of GLS_net_noBackground upon the PV testing set.

Name	Precision	Recall	F1-Score	Support
GLS	35.6	75.5	48.4	143
notGLS	94.7	76.2	84.5	820
Macro Avg	65.2	75.9	66.4	963
Weighted Avg	85.9	76.1	79.1	963
Accuracy	76.1			

## Data Availability

Data is available in the Supplementary Materials.

## Supplementary Materials

The following supporting information can be downloaded at https://www.mdpi.com/article/10.3390/plants11151942/s1: Figure S1: GLS_net performance; Figure S2: GLS_net_pv performance; Figure S3: leafRCNN performance; Figure S4: GLS_net_noBackground performance; Table S1: Detailed leafRCNN performance upon the IFL testing set; Table S2: leafRCNN loss upon the IFL testing set. IF dataset: https://www.kaggle.com/datasets/hamishcrazeai/maize-in-field-dataset (accessed on 21 July 2022) (See Reference [39] for DOI). IFL dataset: https://segments.ai/Hamish_Craze/GLS_instanceSegmentationEasy_leaf/ (accessed on 21 July 2022).
