# Supplementary material for: Deep Learning Diagnostics of Gray Leaf Spot in Maize under Mixed Disease Field Conditions
Source: Plants (Basel). 2022 Jul 26;11(15):1942. doi: 10.3390/plants11151942 (PMC9330607; doi:10.3390/plants11151942)
Supplement: Supplementary file 1 [file plants-11-01942-s001.zip › plants-1748922-supplementary.pdf]

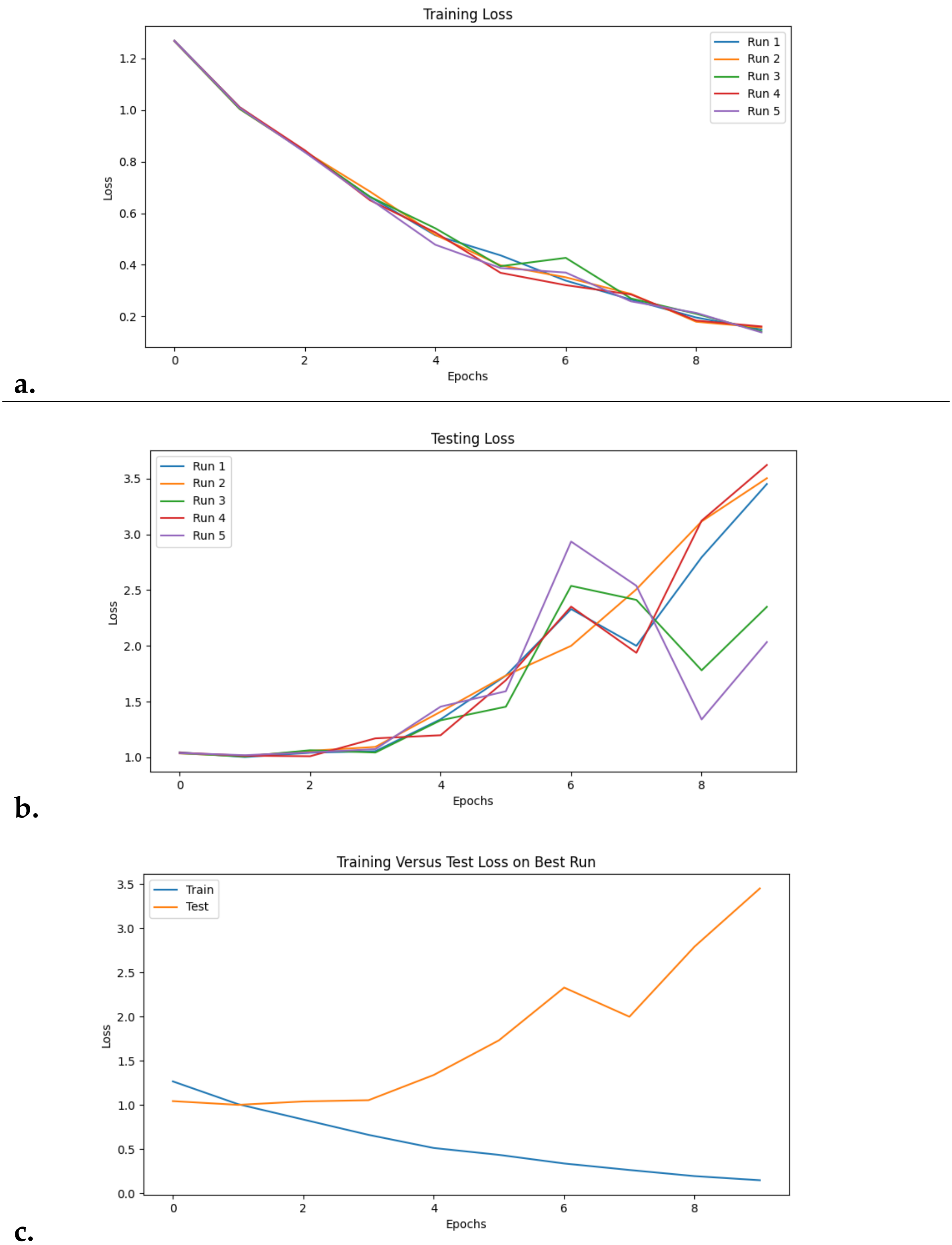


**Figure S1.** GLS_net performance (a) GLS_net upon the IF training set across 5 runs of 10 epochs (b) GLS_net performance upon the IF testing set across 5 runs of 10 epochs. (c) Best GLS_net model performance upon the IF testing and training set across 10 epochs.

**a.**

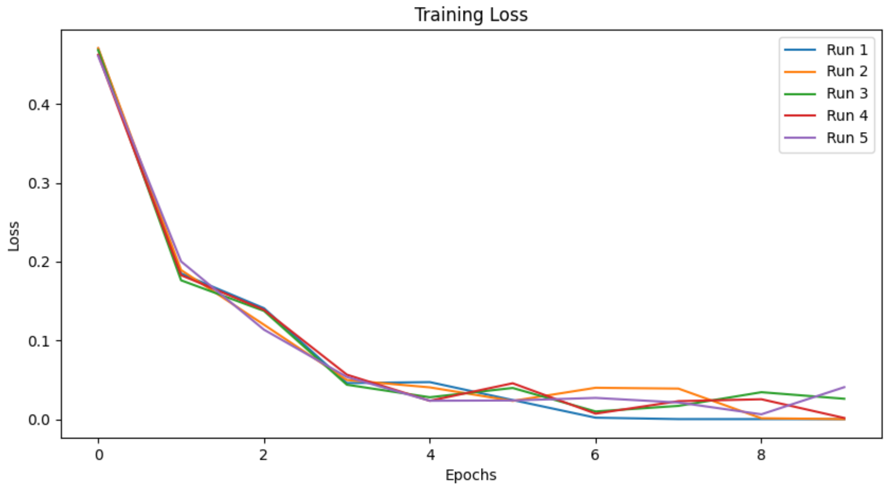


**b.**

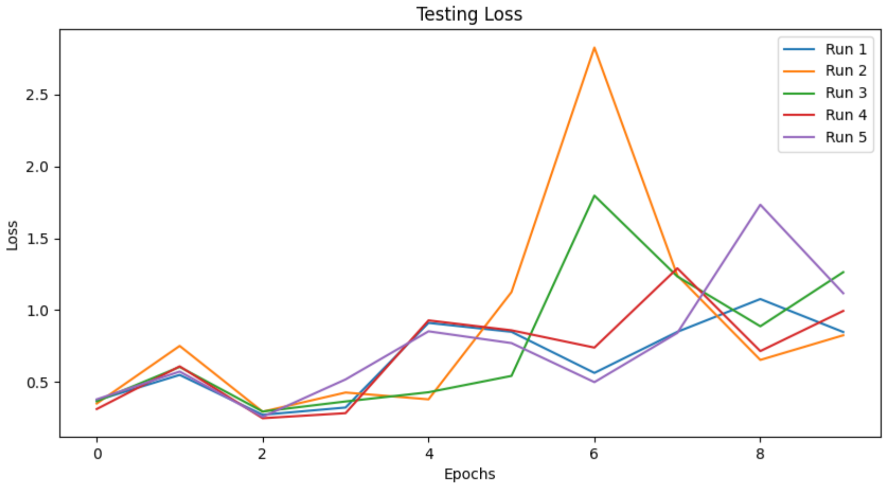


**c.**

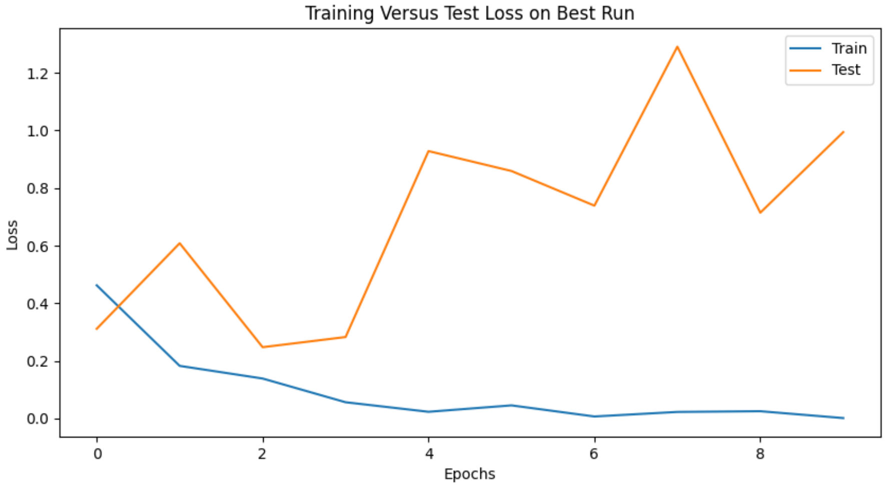


**Figure S2.** GLS_net_pv performance (a) GLS_net_pv performance upon the PV training set across 5 runs of 10 epochs (b) GLS_net_pv performance upon the PV testing set across 5 runs of 10 epochs (c) Best GLS_net_pv performance upon the PV training and testing set across 10 epochs.

Supplementary Datafile: Deep learning diagnostics of gray leaf spot in maize under mixed disease field conditions, Craze et al. Plants 2022

**Table S1.** Detailed leafRCNN performance upon the IFL testing set

| Metric | IoU | maxDets | maxDets | Score |
|---|---|---|---|---|
| **Bounding Box** | | | | |
| Average Precision | 0.50:0.95 | all | 100 | 0.990 |
| Average Precision | 0.50 | all | 100 | 0.899 |
| Average Precision | 0.75 | all | 100 | 0.990 |
| Average Precision | 0.50:0.95 | small | 100 | 0.990 |
| Average Precision | 0.50:0.95 | med | 100 | 0.899 |
| Average Precision | 0.50:0.95 | large | 100 | 0.990 |
| Average Recall | 0.50:0.95 | all | 1 | 0.990 |
| Average Recall | 0.50:0.95 | small | 100 | 0.899 |
| Average Recall | 0.50:0.95 | med | 100 | 0.990 |
| Average Recall | 0.50:0.95 | large | 100 | 0.939 |
| **Segmentation (Mask)** | | | | |
| Average Precision | 0.50:0.95 | all | 100 | 0.923 |
| Average Precision | 0.50 | all | 100 | 1.000 |
| Average Precision | 0.75 | all | 100 | 0.977 |
| Average Precision | 0.50:0.95 | large | 100 | 0.923 |
| Average Recall | 0.50:0.95 | all | 1 | 0.942 |
| Average Recall | 0.50:0.95 | large | 100 | 0.944 |

**Area =** Indicates the area in which AR and AP values were calculated. Since leaves in the IFL dataset are quite large and take up most of the area in a image, only AP and AR values for the entire image are shown here
**maxDets =** The maximum number of predicted instances that will be considered. For example: An image may contain 400 predicted instances of class A. Of these, 100 instances of class A will be randomly selected and assessed. The IFL dataset only contains a single instance in each image.

**Table S2.** leafRCNN loss upon the IFL testing set

| Best Loss* | Average Best Loss* | Best Loss Standard Deviation* |
|---|---|---|
| 0.0571 | 0.062 | 0.0308 |

*Results are from five separate runs of 10 epochs each.

Supplementary Datafile: Deep learning diagnostics of gray leaf spot in maize under mixed disease field conditions, Craze et al. Plants 2022

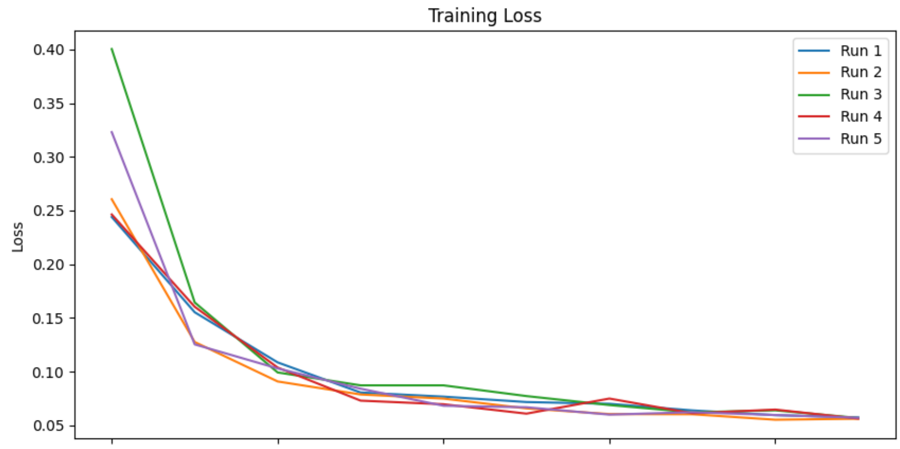


a.

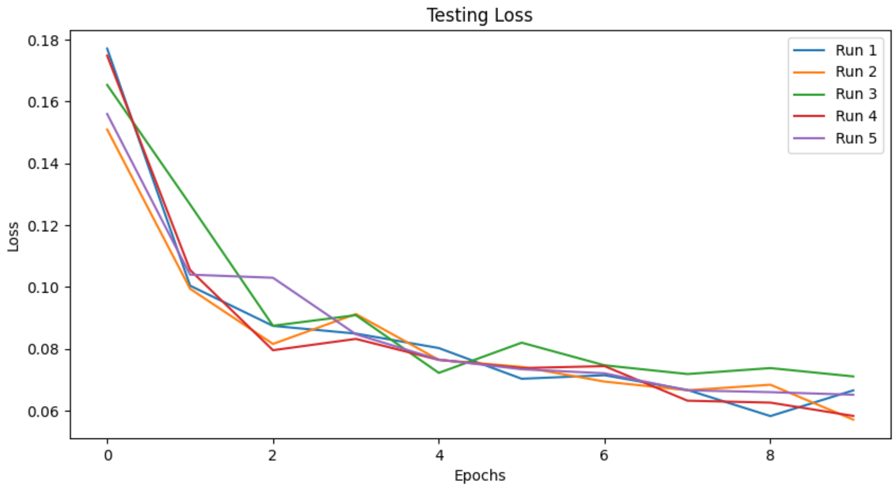


b.

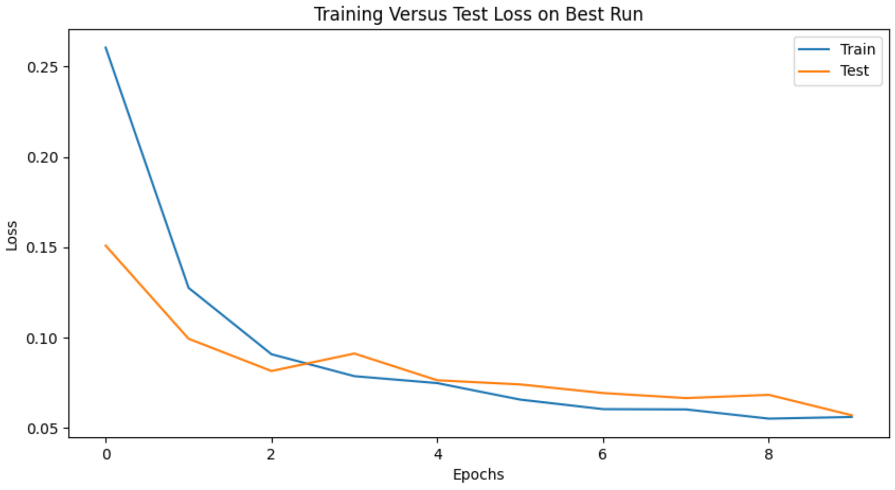


c.

**Figure S3.** leafRCNN performance. (a) leafRCNN performance upon the IFL testing set across 5 separate runs of 10 epochs (b**)** leafRCNN performance upon the IFL training set across 5 separate runs of 10 epochs (c) Best leafRCNN model performance upon the IFL testing and training set across 10 epochs.

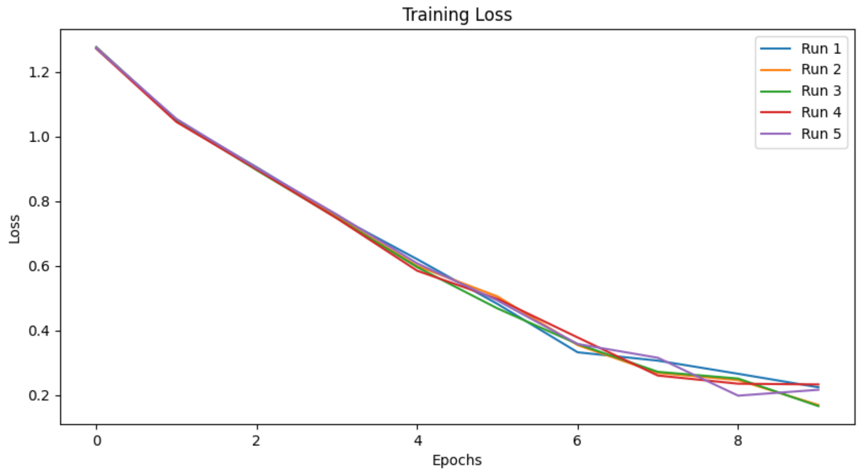


**a.**

**b.**

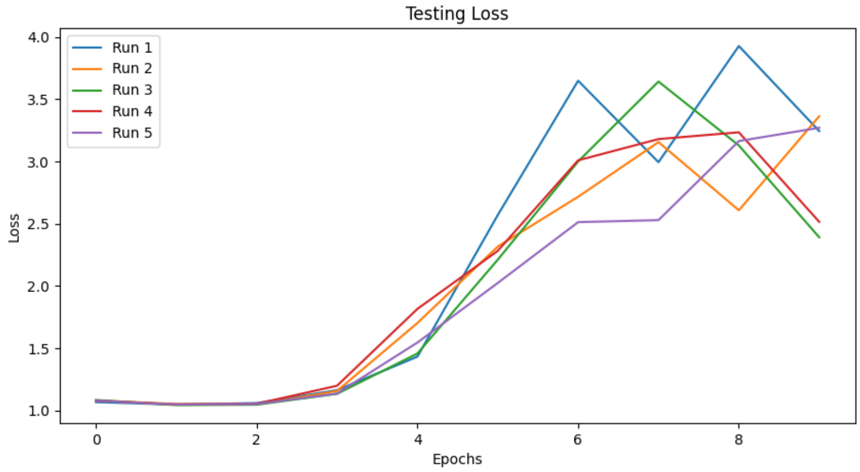


**c.**

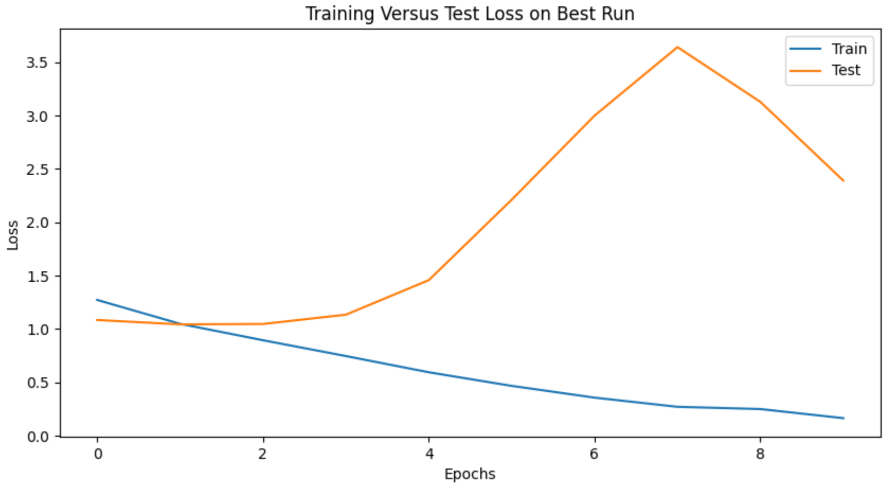


**Figure S4.** GLS_net_noBackground performance (a) GLS_net_noBackground performance upon the IFNB training set across 5 runs of 10 epochs. (b) GLS_net_noBackground performance upon the IFNB testing set across 5 runs of 10 epochs. (c) Best GLS_net_noBackground performance upon the IFNB training and testing set across 10 epochs.
